# Affordable remote monitoring of plant growth in facilities using Raspberry Pi computers

**DOI:** 10.1002/aps3.11280

**Published:** 2019-08-12

**Authors:** Brandin Grindstaff, Makenzie E. Mabry, Paul D. Blischak, Micheal Quinn, J. Chris Pires

**Affiliations:** ^1^ Division of Biological Sciences and Bond Life Sciences Center University of Missouri Columbia Missouri 65211 USA; ^2^ Department of Ecology and Evolutionary Biology University of Arizona Tucson Arizona 85721 USA; ^3^ Division of Information Technology University of Missouri Columbia Missouri 65211 USA

**Keywords:** environmental sensing, growth chamber, Growth Monitor pi (GMpi), humidity, light, Raspberry Pi, temperature

## Abstract

**Premise:**

Environmentally controlled facilities, such as growth chambers, are essential tools for experimental research. Automated, low‐cost, remote‐monitoring hardware can greatly improve both reproducibility and maintenance.

**Methods and Results:**

Using a Raspberry Pi computer, open‐source software, environmental sensors, and a camera, we developed Growth Monitor pi (GMpi), a cost‐effective system for monitoring growth chamber conditions. Coupled with our software, *GMPi_Pack*, our setup automates sensor readings, photography, and alerts when conditions fall out of range.

**Conclusions:**

GMpi offers access to environmental data logging, improving reproducibility of experiments and reinforcing the stability of controlled environmental facilities. The device is also flexible and scalable, allowing researchers the ability to customize and expand GMpi for their own needs.

Growth chambers play an important role in plant science and agronomics by providing and maintaining constant growing conditions in order to reduce variables that could bias experimental data. However, the environmental parameters of growth chambers can fluctuate, which can impede reproducibility in future experiments (Lee and Rawlings, 1982; Potvin and Tardif, 1988). In order to compensate for issues of reproducibility, researchers typically randomize the placement of plants in the growth chamber and perform replications of the experiment (Hammer and Hopper, 1997). To make certain that the environmental parameters inside a growth chamber are within the required parameters, the best solution is to monitor and record them. Monitoring and recording the environmental variables inside a growth chamber can be used to increase repeatability for future experiments and provide researchers with real‐time information about the conditions their plants are experiencing.

Single‐board computers (e.g., Raspberry Pi [www.raspberrypi.org], Orange Pi [www.orangepi.org], Beagle Board [www.BeagleBoard.org], Arduino [www.arduino.cc]) paired with open‐source software provide the opportunity to develop such a system of growth chamber monitoring. The variety of different sensors (e.g., light, temperature, humidity, pH, motion) and single‐board computers afford a high degree of flexibility and can be used in many different applications, such as the internet of things (IoT) and otherwise. IoT devices are internet‐connected objects that are capable of collecting data and sending it through the internet. These devices can also be integrated to build scalable networks of reconfigurable computers capable of environmental monitoring (Ferdoush and Li, 2014) or used for other tasks (e.g., plant phenotyping; Tovar et al., 2018) that can be invaluable in our data‐driven field.

Monitoring systems developed using this technology are most prevalent in agriculture. Two of the most popular applications are selective irrigation and predictive analytics, both of which can improve productivity and efficiency of water management. For example, Shah and Bhatt (2017) used Raspberry Pi computers to monitor the conditions of a greenhouse using temperature, humidity, and soil moisture sensors that autonomously logged data to a cloud server. Raspberry Pi computers can also be used to automate irrigation. Cabaccan et al. (2017) used a network of Raspberry Pi computers equipped with sensors for light, temperature, and humidity, as well as a real‐time clock and external battery, to monitor lettuce plants (*Lactuca sativa* L.). The applications of Shah and Bhatt (2017) and Cabaccan et al. (2017) show the scalability of this technology as well as the ability to run on an independent power source for a period of time, giving needed flexibility for use in places with limited access to electricity. In other cases, Raspberry Pi computers have been used to detect system failures that would otherwise have gone unnoticed (Gurdita et al., 2016). This technology not only enables new means and methods of data collection and plant care, it also offers a cost‐effective way to improve existing monitoring systems through redundancy or improved precision depending on the system.

Using Raspberry Pi as a platform (Halfacree and Upton, 2012; Gay, 2014), we have developed the Growth Monitor pi (GMpi; Fig. 1A–C) to be an affordable way to monitor plants in growth chambers and greenhouses. GMpi stands out from other devices described above because our *GMPi_Pack* software combines multiple features, such as sensing (temperature, humidity, and light intensity), cloud storage, image capture, and alerts, onto a single platform. It relies heavily on software already available from the open‐source community and is meant to illustrate how this technology can be developed and used by researchers who may not be as familiar with software engineering. With the detailed protocol below, we hope that this type of monitoring and sensing can now be made accessible to anyone.

**Figure 1:**
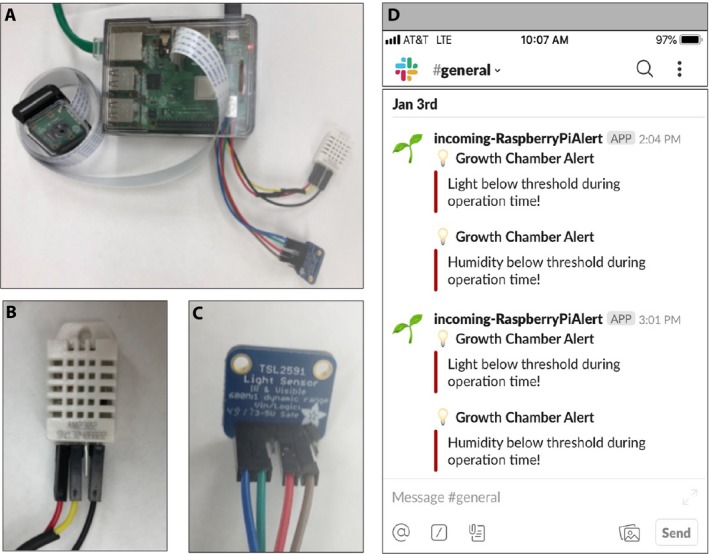
GMpi, sensors, and Slack alert. (A) Photograph of the complete GMpi setup with all peripheries attached. (B, C) Close‐up photos of the temperature and humidity sensor (DHT22) and light intensity sensor (TSL2591). (D) A snapshot of what an incoming alert from the GMpi looks like on Slack. This will alert all members of this workspace that the growth chamber has fallen out of the specified range for both light and humidity.

## METHODS AND RESULTS

### Raspberry Pi and peripheries

The GMpi system was developed using a Raspberry Pi Model B+ single‐board computer installed with the Raspbian Stretch 4.14.50‐v7+ operating system (Raspberry Pi Foundation, Cambridge, United Kingdom). A temperature and humidity sensor (DHT22; Adafruit Industries LLC, New York, New York, USA) was used to collect data about the humidity and temperature inside the growth chamber. DHT sensors are useful as they are equipped with a capacitive humidity sensor and a thermal resistor. The accuracy of the DHT22 sensor was determined by testing its readouts against the thermostat and analog hygrometer that are installed in the growth chamber being used. To collect data about light intensity in both full spectrum and infrared wavelengths, the TSL2591 (Adafruit Industries LLC) sensor was used. This sensor is capable of detecting light between 0.000118 and 88,000 lux (lumens per square meter), allowing us to detect minute changes in light intensity in both the visible and infrared spectra. Output of common fluorescent bulbs are described in lumens rather than photosynthetically active radiation (PAR); therefore, lux was chosen to log lumens in a given area to detect a change in output from fluorescent tubes in our growth chamber, such as light bulbs going out and needing replacement. Finally, a camera was connected to relay visual information to the end user over the internet, allowing for remote observation of plant growth. The GMpi can be assembled and configured for approximately US$200. Compared to other popular commercial solutions for environmental data logging in a large growth facility, such as Argus (Argus Control Systems Ltd., Surrey, British Columbia, Canada; www.arguscontrols.com), which ranges from US$10,000 to more than US$1,000,000, the GMpi is a much more cost‐effective alternative.

### Connecting sensors

**Figure 2:**
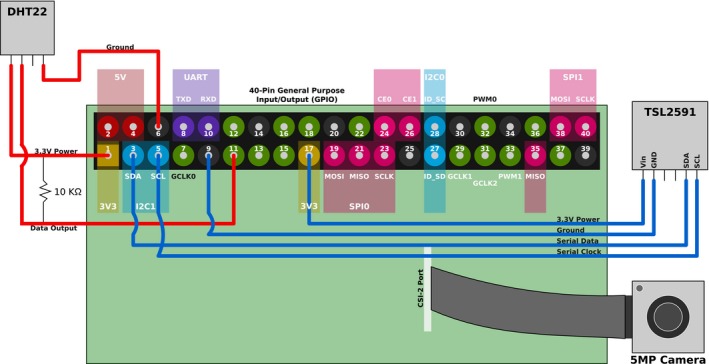
A diagram showing the connections between the Raspberry Pi and the attached peripheries and the Raspberry Pi GPIO pinout. Illustrated are the connections for the light intensity sensor (TSL2591) to the GPIO pins: pin 3 for SDA (serial data), pin 5 for SCL (serial clock), pin 9 for ground, and pin 17 for 3.3‐V power. The diagram also illustrates the connection of the temperature and humidity sensor (DHT22) to the GPIO pins: pin 1 supplying 3.3‐V power, pin 6 for ground, and pin 11 for data. In addition, the figure shows where the resistor should be soldered between the power and data wire for the temperature and humidity sensor (DHT22). Finally, it shows where the ribbon cable from the camera board is connected to the Raspberry Pi.

To connect the different sensors to the Raspberry Pi, expertise in soldering is required. We enlisted help from our department's shop to achieve this. To connect the temperature and humidity sensor (DHT22), the first pin from the right was connected to a 3.3‐V general purpose input/output (GPIO) pin. The second pin was then connected to a data pin, the third was not used, and the fourth was connected to a ground (Fig. 2). A 10‐KΩ resistor was soldered between the power wire and data wire to allow data output (Fig. 2). For the light sensor (TSL2591), the first pin labeled “Vin” was connected to a 3.3‐V GPIO pin, then the pin labeled “GND” was connected to a ground pin (Fig. 2). This device uses an inter‐integrated circuit (I2C) serial protocol in order to communicate with devices by connecting the pins labeled “SDA” (serial data) and “SCL” (serial clock) to pins 3 and 5 of the Raspberry Pi. Finally, the camera was connected to the camera serial interface (CSI‐2) port behind the ethernet port via a ribbon cable. The ribbon cable provides additional flexibility in the placement of the camera for optimal viewing of plants.

### Uploading to a cloud drive service

In order to upload the resulting sensor data to a cloud service, we used a third‐party, open‐source application called Rclone (https://github.com/pageauc/rclone4pi). Rclone can be configured to connect to several popular cloud storage services such as Google Drive, Box, and Dropbox. We elected to use Google Drive because it is a free service. After adjusting the Rclone configuration settings, a link is provided that allows for a connection to the cloud service of choice. Uploading the collected data and images is handled by the *UploadFile()* and *UploadFile2()* functions in *GMPi_Pack.py* library (discussed below). These scripts are called in *packageTest.py*,* picSnap.py*, and *upload.py* files and can be run automatically using the Unix tool cron.

### Remote monitoring

A unique addition to the GMpi is its ability to notify users if a growth chamber or greenhouse falls out of a specified range for temperature, humidity, or light. We took advantage of the popular platform Slack (https://slack.com/), using the incoming webhooks function to set up remote notifications (Fig. 1D). This allows notifications to be sent to a configured Slack channel when thresholds are hit. As the use of Slack to communicate with research groups increases, it makes sense to have equipment be able to communicate via Slack as well. Using Slack incoming webhooks allows for end‐users to configure what messages will generate a notification being sent from Slack to their device(s) that access Slack. It is also possible to “mention” users directly in the Slack webhook message so that specific users get a notification in the channel.

### GMpi software dependencies

The scripts used by the GMpi for the two sensors, temperature and humidity (DHT22) and light (TSL2591), are dependent on the open‐source Python libraries *Adafruit_DHT* (https://github.com/adafruit/Adafruit_Python_DHT) and *python‐tsl2591* (https://github.com/maxlklaxl/python-tsl2591). These libraries enable the device to read and process output from the sensors. The camera uses software that is already packaged with the Raspbian Stretch operating system (I2C). To combine automation, remote monitoring, and data collection, we developed our own software package, *GMPi_Pack*, which is freely available on GitHub (https://github.com/BrandinGrindstaff/GMpi) under a GNU General Public License (v3). *GMPi_Pack* is composed of seven scripts: *GMPi_Pack.py* (the main library), *configuration.py*,* packageTest.py*,* picSnap.py*,* sense.py*,* upload.py*, and *install.sh*. After getting the software from GitHub, users should run the *install.sh* script. This script will attempt to download and install all software dependencies for *GMPi_Pack* automatically. Next, users should run the *configuration.py* script, which sets the minimum and maximum thresholds for light intensity and creates a configuration file allowing the end‐user to easily modify important information needed for the GMpi to operate. The *packageTest.py* script is included for troubleshooting and gives the user a chance to test the device to confirm that the GMpi is running correctly. Three scripts, *sense.py*,* picSnap.py*, and *upload.py*, call functions from the main *GMPi_Pack.py* library to carry out sensor readings, capture images, and upload data to a cloud service, respectively. These scripts are intentionally independent to allow for modularity when scheduling “cron jobs” to automate these processes. Cron jobs are set up using the Unix cron utility, a time‐based job scheduler that comes packaged with Raspbian Stretch and other Unix‐like operating systems. This allows the user to run shell commands on a time‐based schedule, making it possible to run scripts automatically at user‐set intervals.

### Protocol feasibility

The protocol in Appendices Raspberry Pi hardware setup., Raspberry Pi initial setup., Rclone and GMpi software installation protocol. provides detailed instructions for setting up the GMpi and *GMPi_Pack*, including networking, connecting the hardware, and installing the software. Networking will vary greatly between institutions, and it is advisable to work with an information technology department to work around firewalls that make remote monitoring and secure shell (SSH) access difficult. Both sensors utilized by the GMpi also require soldering before use. The light sensor (TSL2591) requires that the pin headers be soldered onto its printed circuit board, and the temperature and humidity sensor (DHT22) requires a 10‐KΩ resistor be soldered between the power pin and data pin. The software we developed for the Raspberry Pi is dependent on open‐source tools that are available from GitHub and Adafruit, as well as other online sources. Our software, and all of its dependencies, can be downloaded from GitHub (https://github.com/BrandinGrindstaff/GMpi) and set up using the scripts we have provided.

**Figure 3:**
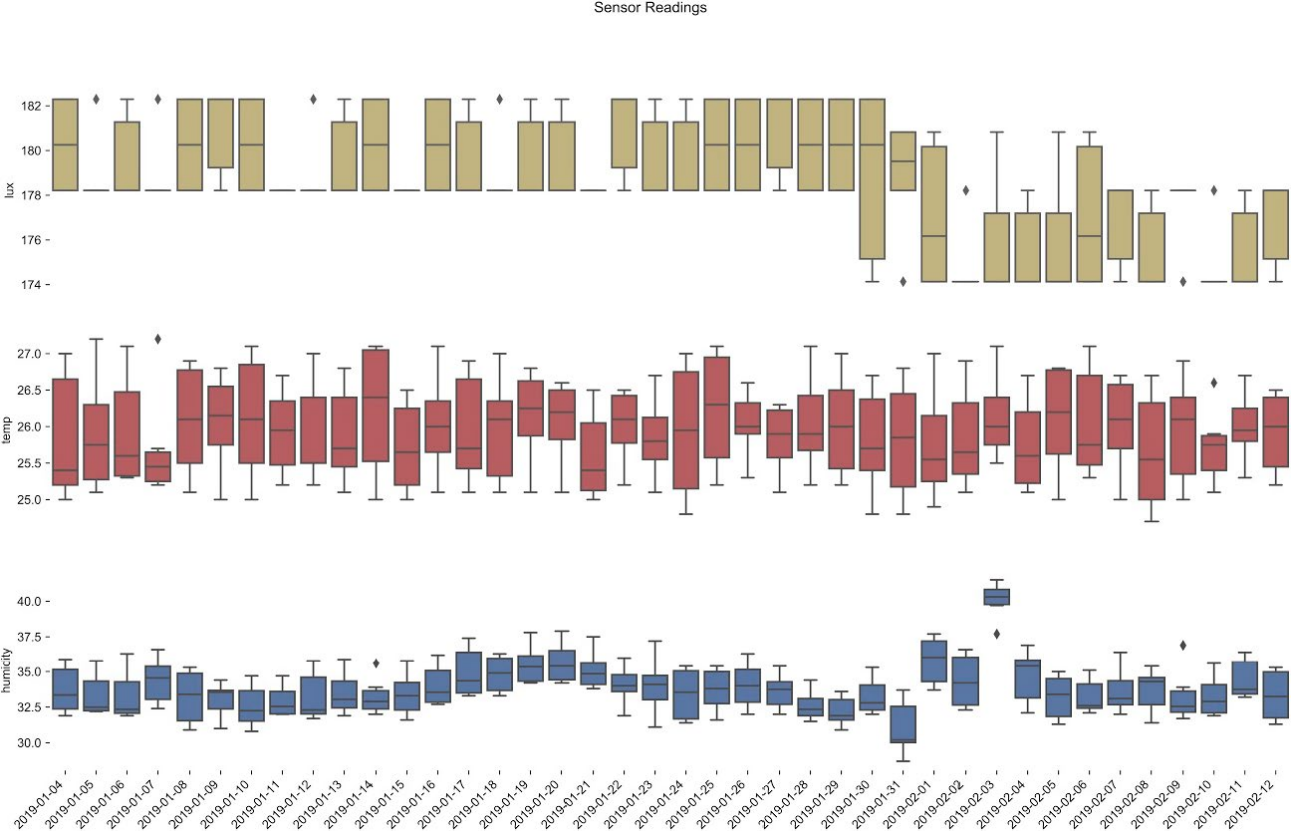
Plot of sensing data, showing approximately one month of data captured with the GMpi. The graph displays light intensity (yellow, top graph), temperature (red, middle graph), and humidity (blue, bottom graph) in box plot format.

To remotely alert users if the chamber or greenhouse falls below desired thresholds, the GMpi uses webhooks to enable it to send alerts through the Slack application. As many groups are now communicating through Slack, we think this will provide a quick way for all members of a research program to be informed that a system is failing. We also provide an example script that will plot output sensor data so users can visually assess the conditions of the environment they are monitoring (Fig. 3). The camera on the GMpi can be coupled with open‐source software, such as plantCV (Fahlgren et al., 2015; Abbasi and Fahlgren, 2016; Gehan et al., 2017; Berry et al., 2018), to allow for plant phenotyping similar to the Bellwether phenotyping platform developed by Fahlgren et al. (2015) or the Raspberry Pi–based system by Tovar et al. (2018). Using the camera in combination with image uploading to cloud storage can also enable the user to diagnose disease or other issues with specimens in the growth chamber remotely from anywhere with internet access. Scalability of the GMpi can also be easily achieved by collecting and storing data across multiple, independent Raspberry Pi devices using unique identifiers for each one. These data can then be accessed through a common cloud storage folder and integrated to generate a more comprehensive view of growing conditions within a facility.

## CONCLUSIONS

The GMpi gives researchers access to a low‐cost option for environmental data monitoring and logging that can improve reproducibility of experiments and reliability of growth chamber conditions, as well as build large data sets that can be employed as phenotypic or environmental data in future studies. With a wealth of free, cost‐effective, and open‐source resources at hand, researchers are in an excellent position to leverage these tools to revolutionize plant science and improve reproducibility in experimentation with little impact on their budgets.

## Data Availability

All script source codes for installing and setting up the GMpi can be downloaded from GitHub (https://github.com/BrandinGrindstaff/GMpi).

## Raspberry Pi hardware setup.

The information provided here covers the physical setup of the Raspberry Pi and its peripheries, including the physical connection of the peripheries. With the exception of connecting the Raspberry Pi to a monitor, the device should be powered off during every step of hardware setup.

**Equipment needed:**

Components ordered from Adafruit Industries LLC (New York, New York, USA; www.adafruit.com):

- Raspberry Pi computer
  ° Raspberry Pi Model 3B+ (1.4‐GHz cortex, A53 with 1 GB RAM; product ID: 3775)
  ° Adafruit Raspberry Pi B+ /Pi 2/Pi 3 case, smoke base (product ID: 2258)
  ° 16 GB SD, MicroSD memory card (16 GB Class 10; product ID: 2693)
  ° 5‐V 2.4‐A switching power supply with 20 AWG MicroUSB cable (product ID: 1995)
- Temperature and humidity sensor
  ° DHT22 temperature–humidity sensor (product ID: 385)
- Light sensor
  ° Adafruit TSL2591 High Dynamic Range Digital Light Sensor (product ID: 1980)
- Camera
  ° Raspberry Pi Camera Board v2 (8 Megapixels; product ID: 3099)
  ° Adafruit Raspberry Pi Camera Board Case with 1/4″ Tripod Mount (product ID: 3253)
  ° Flex Cable for Raspberry Pi Camera (2 meters [if longer cable is desired]) (product ID: 2144)

Other important components that can be purchased elsewhere:

- Female–female 2.54–2.0‐mm multi‐colored jumper wires
- A computer with an SD card reader and internet access
- A monitor with an HDMI port for initial setup
- A keyboard and mouse that use a USB port to interface with the Raspberry Pi during initial setup
- An HDMI cable
- An antistatic wrist strap to protect your circuit boards from static discharge while handling
- Soldering iron
- Heat shrink tubing or electrical tape

### Connecting sensors and other peripheries to the Raspberry Pi

The light intensity sensor (TSL2591) and the temperature and humidity sensor (DHT22) can be interfaced with the Raspberry Pi via generalized purpose input/output (GPIO) pins. The GPIO pins are the 40 pins that run along the upper part of the single‐board computer. The GPIO pins can deliver 3.3 V or 5.0 V of electricity, ground, and data transfer. The data transfer pins include both inter‐integrated circuit (I2C) and universal asynchronous receiver‐transmitter (UART) protocols, although the latter is not used on the GMpi.

*TSL2591 light intensity sensor*

1. Requires the included 6‐pin header to be soldered to the device.
2. Connect female–female jumper wires to pins labeled on sensor “Vin,” “GND,” “SDA,” and “SCL” using a different color for each to distinguish between them easily when connected to the Raspberry Pi. We suggest using white or red for “Vin,” the power‐in connector, and black or brown for “GND,” the ground. This coloring scheme is standard convention, like the battery connectors of a motor vehicle.
  - Connect the serial data (SDA) wire to GPIO pin 3 on the Raspberry Pi (Fig. 2) and connect the serial clock (SCL) wire to GPIO pin 5. These two pins facilitate data transfer for this sensor which uses I2C serial protocol.
  - Connect the ground wire (“GND”) to GPIO pin 9 and the power wire to GPIO pin 17, giving 3.3 V of power to the sensor and grounding it.

*DHT22 temperature–humidity sensor*

1. Connect jumper wires to the power, data, and ground pin, using a different color for each (same as above; adhering to the convention for power and ground if desired). Facing the grid upward (front‐up), the pins are, from left to right, power, data, an unused pin, and ground.
2. A 10‐kΩ resistor must be soldered between the power jumper wire and the data jumper wire in order to transmit data from the temperature and humidity sensor (DHT22) to the Raspberry Pi. Burn a small area of the wire casing off both jumper wires using a lighter. Solder the 10‐kΩ resistor between the power and data wire (Fig. 2).
3. Cover the resistor and exposed wire with heat shrink wire sheathing. Heat the wire sheathing with a wire until it fits snugly. Alternatively, it is also acceptable to use electrical tape as long as the circuit is insulated to prevent shorting.
4. Connect the power wire of the temperature and humidity sensor (DHT22) to GPIO pin 1, the ground wire to pin 6, and the data wire to pin 11 of the Raspberry Pi (Fig. 2).

*Camera and camera circuit board*

1. Connect the camera circuit board to the CSI‐2 port in the lower middle of the Raspberry Pi (Fig. 2). Pull up the black tab on the port to insert the ribbon cable. Close the tab by pressing down on it.
2. The camera listed above has a key to manually focus the camera. We suggest to wait to focus the lens of your camera until your GMpi is completely set up and your camera is in its final fixed position.

*MicroSD card*

1. The microSD card can be inserted into the slot on the bottom left of the Raspberry Pi. However, be sure to install the Raspbian Stretch operating system following the steps outlined in Appendix 2 before inserting the microSD.

*HDMI*

1. Connect the HDMI cable between the Raspberry Pi and an HDMI‐compatible monitor for the initial setup. After the initial setup of the GMpi is complete, it can be accessed without a physical connection using the secure shell (SSH) protocol.

## Raspberry Pi initial setup.

The information provided here covers how to install the Raspbian Stretch operating system onto the Raspberry Pi as well as how to configure the device to use the *GMPi_Pack* software. This section also describes how to connect the Raspberry Pi to a network, which will be different for every user depending on the network being used.

### Raspberry Pi Operating System installation

(Version 4.14 of Raspbian Stretch was used.)

1 Download “Raspbian Stretch with Desktop” from https://www.raspberrypi.org/downloads/raspbian/ (note: downloads as a .zip file).
2 Download “etcher” from https://etcher.io.
  - Select “Download for Windowsx64” (otherwise download the version compatible with your operating system).
3 Set up Etcher software.
  - Accept license agreement and install.
4 Run etcher software; select “year‐month‐day‐raspian‐stretch.zip” (the date will be the date this version was released).
  - Select the SD card you plan to use.
  - Select the flash action and wait for the process to finish.
  - Safely remove the SD card from your computer.
5 Plug in microSD card into your Raspberry Pi.
  - Boot up (external monitor required for this step).

### Configuring Raspberry Pi

(Instructions assume the Raspberry Pi is connected to a monitor, not accessed via SSH.)

1 Select raspberry symbol in the top left of the screen.
2 Scroll down to preferences.
3 Select “Raspberry Pi Configuration.”
4 Click the interfaces tab, and enable:
  - SSH, to connect wirelessly.
  - I2C, for the light sensor.
  - Camera, to use the camera.
  - Remote GPIO, to use our sensors over the internet.
5 Click Localization tab.
6 Select “Set WiFi Country” and scroll down to select the appropriate country.
7 Select “Set Time Zone” and select the appropriate time zone.
8 Select “Keyboard” and select the corresponding locality/language that matches the appropriate keyboard configuration.
9 Click “OK” and reboot the Raspberry Pi.

### Networking

In order to connect to the GMpi device remotely, use an HDMI compatible monitor, a mouse, and a keyboard for the initial configuration to enable SSH access as outlined in the “Configuring Raspberry Pi” section of Appendix 2. With the device configured to use SSH, open a terminal window, and enter the following command:
ifconfig

This command will display your network information in the terminal window. In the ‘wlan0’ section (the third block of text), look for the local IP address labeled ‘inet.’ If your network has dynamic internet protocol (IP) addresses, the address will change on a regular basis, requiring you to repeat this process with the new IP address. Therefore, setting up a static IP address is highly recommended. Universities also tend to have advanced network security protocols. Configuring the device may therefore also require advanced configuration changes to connect to the device wirelessly. For these reasons, it is recommended to contact your institution's information technology department and request a static IP address, or other additional assistance with this step.

## Rclone and GMpi software installation protocol.

This section covers installation of the Rclone software that gives the GMpi the ability to upload collected data to a cloud storage server as well as the installation of the *GMPi_Pack* software. It also covers the use of cron, a time‐based job scheduling software included with the Raspbian Stretch operating system.

### Installing and configuring Rclone for file uploading

For detailed installation information, access the Rclone developer's GitHub (https://github.com/pageauc/rclone4pi). The installation protocol is based on the instructions provided by the developer.

1. Open the terminal and install Rclone.
  - curl ‐L https://raw.github.com/pageauc/rclone4pi/master/rclone-install.sh | bash
2. Upgrade to the most recent version.
  - cd ˜/rpi‐sync
  - ./rclone‐install.sh upgrade
3. Configure Rclone.
  - rclone config
  - A list of options will be given. Select ‘new remote’ by entering “n”.
  - Provide a name for the newly created remote sync location. It is important to remember this name because it is required information for configuring the *GMPi_Pack* software.
  - A list of file sharing services will then be given (e.g., Google Drive, Box, Dropbox). Select the desired service. We decided to use the Google Drive service.
  - <press enter> #this field is for locking program locally to a user name.
  - <press enter> #this field is for setting a password to use this program locally.
  - Select the desired level of access given to the selected cloud account. Full access is recommended, which can be selected by entering “1”.
  - A list host of options will then be given. Pressing “Enter” without any other inputs will accept the default options.
    - y #use autoconfig
  - <press enter>
  - The last input will make the program open a browser and requires the user to login to the chosen cloud service.
    - Log in and select “allow” for Rclone to run. It will ask you if a team drive is being used. Select “y” or “n”.
  - Finally, the program will give an overview of the selected choices. Be sure to look for any errors. If everything looks correct, type “y”.
  - Exit the Rclone configuration by typing “q”.

### *GMPi_Pack* automatic software setup (recommended)

Using the *install.sh* script, the user is able to automatically download all libraries and software for the GMpi. If the user chooses to manually download the *GMPi_Pack* software and its dependencies, follow the steps in the “*GMPi_Pack* manual software setup” section. Otherwise, skip to the section titled “Scheduling sensor readings with cron” after completing the steps below.

1. git clone https://github.com/BrandinGrindstaff/GMpi.git
2. cd GMpi/
3. bash install.sh
4. python3 configuration.py <dht> <data‐pin>

The <dht> and <data‐pin> options for the *configuration.py* script should be substituted with the DHT model (i.e., 22) and pin number where the data sensor is attached to the Raspberry Pi.

After running the *configuration.py* script, a new file named ‘config.txt’ will be generated. Open this configuration file and replace all values listed as ‘<REPLACE>‘ with the desired values used for monitoring (e.g., times for when lights are on/off). The ranges specified here will also be used for alerting the user when growth chambers or greenhouses fall out of desired ranges for sensing.

### *GMPi_Pack* manual software setup

The *install.sh* script included with GMpi will install all software automatically. However, if users wish to install the dependencies separately, the necessary steps to accomplish this are provided below. Use the following commands to download the *GMPi_Pack* software from GitHub and create the configuration file ‘config.txt’.

1. git clone https://github.com/BrandinGrindstaff/GMpi.git
2. cd GMpi/
3. python3 configuration.py <dht> <data‐pin>

Follow the next two sets of instructions to manually install dependencies.

*Humidity and temperature software setup*

For more information, go to https://github.com/adafruit/Adafruit_Python_DHT.

1. Download/install *Adafruit_Python_DHT* library for the humidity and temperature sensor.
  - Open the terminal and download file.
    - git clone https://github.com/adafruit/Adafruit_Python_DHT.git
  - Move to the software folder.
    - cd Adafruit_Python_DHT/
  - Install the software.
    - sudo python3 setup.py install
  - If errors are encountered, try updating the system.
    - sudo apt‐get update
    - sudo apt‐get upgrade
2. Reboot the Raspberry Pi to allow the update to be recognized.
  - sudo reboot
3. If Python v3 is not installed, install it using the following command.
  - sudo apt‐get install build‐essential python‐dev python ‐open ssl

*Light sensor software setup*

For more information on installation and setup, see https://github.com/maxlklaxl/python-tsl2591 and https://learn.adafruit.com/adafruits-raspberry-pi-lesson-4-gpio-setup/configuring-i2c for configuring I2C.

1 Install dependency *libffi* to resolve compatibility issues with Python 3.
  - sudo apt‐get install libffi‐dev
2 Configure I2C (for light sensor).
  - sudo apt‐get install ‐y python‐smbus
  - sudo apt‐get install ‐y i2c‐tools
  - sudo reboot
3 Download/install python‐tsl2951 library for light sensor.
  - git clone https://github.com/maxlklaxl/python-tsl2591.git
  - cd python‐tsl2591/
  - sudo python3 setup.py install

### Scheduling sensor readings with cron

The cron utility is a job scheduling software included in most distributions of Unix‐like operating systems that is included with Raspbian Stretch. It uses the time that is set on the Raspbian Stretch operating system (displayed in the bottom right of the screen, at the far‐right end of the task bar) to determine when to run a program. Cron can be extremely useful for automating processes to run on a set time interval. The GMpi uses cron to execute sensor reads, capture images, and upload data at set time intervals without manual execution by the user.

- To create a crontab file, execute the following commands in a terminal window.
  - crontab ‐e
    - This will give a set of options for selecting a text editor to make changes to the crontab file. Select the desired text editor (we chose nano) and begin editing your crontab file.
    - A crontab entry accepts six fields separated by spaces.
      1. From left to right: minute, hour, day of the month, month, day of the week, and the command to be executed.
    - There are also a number of functions that can be done with integers in any of those first five fields, as long as they are within the specified range of the field described in the text at the top of the file.
      1. To denote “first through last” in one of the first five fields use an asterisk.
    - Crontabs invokes the command from the user's HOME directory, so it is important to include the correct file path in your command.
      1. For example:
        - */2 * * * * cd ˜/GMpi && python3 sense.py
          - This tells crontabs that every other minute (*/2), of every hour (*), of every day of the month (*), of every month (*), and every day of the week (*), to change directory (cd) from user's HOME folder to the GMpi (˜/GMpi) folder and (&&) to use python3 to run “sense.py” and collect sensor readings.
      2. For more information on setting up cron jobs, read the text at the top of the created crontab file and/or read the manual to crontab or cron included with the Raspbian Stretch operating system.

### Remote notifications

To create an incoming webhook for Slack notifications, first choose the desired workspace for receiving alerts. It is recommended that users create a separate GMpi channel to facilitate communication. Once a workspace has been chosen, install a new app to the workspace by selecting “+ add apps” at the bottom of the left‐most column. Search for the “Incoming Webhooks” option and click “Install.” Once installed, users can modify the settings to choose which channel GMpi should post to, add descriptive labels, and customize names and icons. The webhook URL link included in the settings section is what needs to be added to the configuration file for alerts to be sent successfully.
